# Reduction of Donor Site Morbidity of Free Radial Forearm Flaps: What Level of Evidence Is Available?

**Published:** 2012-02-03

**Authors:** Denys J. Loeffelbein, Sammy Al-Benna, Lars Steinsträßer, Robin M. Satanovskij, Nils H. Rohleder, Thomas Mücke, Klaus-Dietrich Wolff, Marco R. Kesting

**Affiliations:** ^a^Department of Oral and Maxillofacial Surgery, Technische Universität München, München, Germany; ^b^Department of Plastic Surgery and Burn Center, BG University Hospital Bergmannsheil, Ruhr-University Bochum, Bochum, Germany

## Abstract

**Background:** The radial forearm free flap (RFFF) is the most commonly used free flap in head and neck reconstructive surgery. However, despite excellent results with respect to the site of reconstruction, donor site morbidity cannot be neglected. This review summarizes the current state of knowledge and analyzes the level of evidence with regard to perioperative management of the reduction of RFFF donor site morbidity. **Methods:** The medical Internet source PubMed was screened for relevant articles. All relevant articles were tabulated according to the levels of scientific evidence, and the available methods for reduction of donor site morbidity are discussed. **Results:** Classification into levels of evidence reveals 3 publications (1.5%) with level I (randomized controlled trials), 29 (14.0%) with level II (experimental studies with no randomization, cohort studies, or outcome research), 3 (1.5%) with level III (systematic review of case-control studies or individual case-control studies), 121 (58.7%) with level IV (nonexperimental studies, such as cross-sectional trials, case series, case reports), and 15 (7.3%) with level V (narrative review or expert opinion without explicit critical appraisal). Thirty-five (17.0%) articles could not be classified, because they focused on a topic other than donor site morbidity of the RFFF. **Conclusions:** Although great interest has been expressed with regard to reducing the donor site morbidity of the workhorse flap in microvascular reconstruction procedures, most publications fail to provide the hard facts and solid evidence characteristic of high-quality research.

Postablative head and neck cancer reconstructive surgery frequently requires the replacement of tissue to provide the most functional and aesthetic result. Microvascular free flaps have the advantage of providing healthy vascularized nonirradiated tissue for recipient sites that may have been compromised by surgery, radiation, chemotherapy, or a combination of all 3. Since its introduction by Yang et al^1^ in 1981, the fasciocutaneous radial forearm free flap (RFFF) has become the most commonly used free flap in postablative head and neck reconstruction.^2^^-^4 Its advantages include its thinness, versatility, and pliability, the relative hairlessness of volar wrist skin, and the reliability of its long and large-diameter pedicle, making it suitable especially for the replacement of the intraoral mucosa but also other application fields such as the reconstruction of complex hand defects.^5^^-^7 Unfortunately, one “robs Peter to pay Paul” by using this flap. Although excellent closure results of the primary defect can be achieved, a concomitant functional and aesthetic morbidity of the weakened donor site is often observed. Over the last few decades, the modifications that have been achieved in an effort to improve these undesirable features include various types of dissection techniques, autologous or artificial skin grafts, primary closure procedures, or switching to comparable free flaps.

This article reviews current evidential knowledge of the earlier named topics and perioperative aspects with regard to the RFFF donor site.

## METHODS

We used the PubMed (www.pubmed.gov) electronic information sources and database. The last electronic update was carried out on October 5, 2011, with the key words: “donor site morbidity” and “radial forearm flap” within the “all fields” search builder, including title and abstract, if available. The scientific quality of each identified contribution was evaluated by the first author (D.J.L.) and the last author (M.R.K.) by using a modified version of the Antes classification for the levels of evidence in therapy^8^ (Table 1). Articles with the major topic of donor site morbidity of the RFFF, which were not found during database research, but were known by the authors or found during preparation of this article were included. Articles with a main focus on a topic other than donor site morbidity of the free radial forearm flap were excluded.

We classified the publications that included meta-analyses or individual randomized controlled trials (RCTs) as level I, individual cohort studies, experimental studies with no randomization, and quality-of-life research as level II, case-control studies as level III, nonexperimental studies and case reports as level IV, and expert opinion as level V. To differentiate between publications classified within evidence level IV, we categorized them according to the number of participants (IVa > 100; IVb 20-100; IVc < 20). Level VI, which is not a level of evidence, recorded articles that were not classifiable.

## RESULTS

The computer-assisted search of the Internet PubMed database yielded 188 hits. In addition, 18 relevant articles were found, which were not identified by the database, during preparation of the manuscript. All articles (n = 206) were tabulated according to the levels of scientific evidence. After evaluation of the manuscripts, or if not available, the abstracts, 35 contributions were classified as irrelevant to the discussed topic and were recorded as level VI.

No systematic review of RCTs was published during the period covered by our search (evidence level Ia).

Three contributions (1.5%) were articles about RCTs (evidence level Ib). Two of these papers focused directly and solely on the improvement of donor-site morbidity: Moazzam and Gordon^9^ recommended cross-suturing as an aid to wound closure in order to reduce the size of any full-thickness skin defect before skin grafting and saw fewer complications such as skin graft failure and tendon adherence with their technique. Meland et al^10^ investigated the donor site morbidity of the osteocutaneous RFFF (ORFFF) in an animal trial, utilizing sheep tibia, and found an unacceptable increase in the weakness of the bone. Even a one-quarter diameter bone removal resulted in significant reduction to only 26% of original strength and to 29% of original stiffness (resistance to deformation). They recommended the use of other revascularized bone transplants for bony reconstruction.

Although the radius bone is significantly weakened by the harvest of a graft, a level IIb study of Edmonds et al^11^ has shown that much of this strength can be regained with plate fixation of the radius. Plating techniques such as direct plating^12^^,^^13^ (both evidence level IV) were developed to reduce the risk of postoperative fracturing. This lends further credibility to the ORFFF as a safe and reliable source of vascularized compound osteocutaneous flaps for reconstructive procedures in nonstressed regions, for example, for total nasal defect reconstruction. However, for those force-exposed areas such as mandible or maxillofacial defects, other more robust transplants are available in our opinion.

The third level Ib study by Chau et al^14^ determined which method of fascial dissection and skin graft reconstruction of RFFF defects had superior functional and cosmetic outcomes. They compared the suprafascial dissection technique with meshed or sheet graft reconstruction to the subfascial dissection technique with meshed or sheet graft reconstruction. The functional, cosmetic, and tendon exposure outcomes were collected prospectively and blindly analyzed by validated self-report questionnaires and objective functional measurements. The suprafascial dissection with sheet graft reconstruction yielded superior functional, cosmetic, and tendon exposure outcomes to those of the other groups.

Twenty-nine contributions (14.0%) were identified having evidence level II: none in level IIa, 8 in level IIb, and 21 in level IIc. Three papers (1.5%) were recognized as having evidence level III; all of them were in level IIIb. One hundred twenty-one contributions (58.7%) were identified as having evidence level IV and 15 (7.3%) with level V. Thirty-five (17.0%) articles could not be classified, because they focused on a topic other than that of donor site morbidity of the RFFF and were therefore classified as level VI (Table 2).

All relevant articles mainly focused on 3 topics, namely (1) the flap raising techniques of the RFFF, (2) donor site closure methods, and (3) the comparison of donor site morbidity to other free flaps.

### Flap raising

Research in suprafascial preparation or prelamination techniques of the RFFF, together with donor site morbidity, is mainly based on retrospective studies. Only 2 prospective studies investigated and compared different dissection techniques with different reconstructive methods: first, the earlier discussed, prospective, randomized controlled level Ib study by Chau et al^14^; second, Lutz et al^15^ have shown, in a prospective, but unfortunately not controlled, designed outcome research (thus evidence level IIc) in a large series of 95 consecutive cases, the advantages of the suprafascial elevation technique to the classic elevation technique. Particularly with regard to a higher rate of immediate complete successful skin grafts, the impairment of the range of motion and strength of the donor hand was avoided. Other studies with less evidence support these ideas, as the deep fascia protects the flexor tendons,^16^^-^19 and the entire donor site remains covered with the well-vascularized deep fascia, thus preventing the exposure and tenting of the flexor tendons, a problem that is related to minor donor site morbidity. In the majority of cases, this technique results in improved donor site outcome but, frequently, at the expense of the viability of the flap^20^^,^^21^ (all evidence level IV).

One further option during flap raising to decrease postoperative complications, namely, the exposure of the flexor carpi radialis tendon and possible consecutive skin graft failure, is to mobilize the belly of the flexor digitorum sublimis muscle to lie over to that of the flexor pollicis longus muscle, so that the flexor carpi radialis tendon is covered. This technique, described in 1985, eliminates tendon exposure but can provoke a compression of the median nerve^22^ and has therefore been modified by Swift et al^23^: the median nerve, which is frequently adherent to the underside of the flexor digitorum sublimis muscle belly, is mobilized and displaced, and thus, its compression by the overlying structures is prevented (both evidence level IV).

Another idea to reduce donor site morbidity and to avoid a second donor site is the prelamination of the RFFF with autologous^24^ (evidence level IIc) or tissue-engineered oral mucosa^25^ or a split-thickness skin graft (STSG)^26^ (both evidence level IV). This 2-step procedure, which elongates preoperative time, must first be ethically considered in cancer patients. Furthermore, one must be aware of secondary shrinkage of the flap. The preservation of skin and subcutaneous tissue of the forearm enables primary wound closure, further reducing donor-site morbidity. Unfortunately, high-level studies are missing to encourage further research into this promising field in order to produce better evidence and further improvements of donor and recipient sites in reconstructive head and neck surgery.

The authors recommend that the flap is raised in the classic secure subfascial way on the nondominant arm. The use of a tourniquet is not mandatory in our opinion and even prolongs the ischemia time. When transecting the fibrous attachments between the undersurface of the forearm fascia and the paratenon, the paratenon that envelops the muscle tendons must crucially be left untouched to achieve an accurate wound bed. Extensive mobilizing and oversewing of the surrounding muscle bellies are not necessary and not recommended. The flap should not be extended to the dorsal aspect of the arm for aesthetic reasons. Opinions differ, if an integration of the cephalic vein increases donor site morbidity^27^ (evidence level V). However, the superficial branch of the radial nerve must be identified over the paratenon of the brachioradialis muscle and must be carefully preserved during further dissection; otherwise, paresthesia of the back of the hand, including the web of skin between the thumb and index finger, could occur. The positioning of the distal margin more proximally might prevent exposure of the tendon of the brachioradialis muscle. For exposure of the proximal vascular pedicle, a wavy-line incision helps reduce postoperative scar shrinkage.

### Closure of the donor site

#### Direct closure

Direct closure of the radial forearm flap donor site is commonly considered as the method of choice if possible; it avoids the complication of delayed wound healing, but its application is restricted to narrow wounds. If the donor site defect exceeds a range of 2 to 3 cm (depending on the elasticity of the tissue), direct closure is not possible without special approaches. An evidence level Ib study by Moazzam and Gordon^9^ describes a cross-suturing technique as an easy method for the reduction of RFFF donor sites when direct closure is not possible. In this prospective randomized study including 20 patients, a smaller graft is needed for closure, and fewer complications such as skin graft failure and tendon adherence to graft have been seen.^9^

From other methods for the reduction and closure of the skin defect all over the body, only the “purse-string” suture has been researched, on an evidential basis, in cases of the RFFF donor site defect. This technique has been evaluated by Winslow et al^28^ in a level IVb study for the treatment of RFFF donor sites. During this procedure, a suture is made in a circumferential fashion, avoiding the inclusion of superficial branches of the radial nerve. As the purse string is tightened, the size of the primary defect is reduced, and the remaining defect can be covered with a smaller skin graft. In this prospective, unfortunately not controlled, study, the authors treated the donor sites of 67 patients with a purse-string prior to skin grafting. A mean decrease in the defect size of 44.5% and significantly improved aesthetic outcomes could be achieved.^28^

#### Local flaps

Local skin flaps are adjacent to the defect margin and can be considered for RFFF donor site closure, when defect size is limited, and when the elasticity of the surrounding tissue is sufficient.

The Z-plasty technique has been described for the RFFF donor site closure by Hui et al^29^ in 1999 (evidence level IVc). It is based on a Z-shaped incision, which generates 2 opposed triangular flaps that are reunited after transposition, thus elongating the tissue and allowing defect coverage. However, although this method has distinct advantages, its application has not been reported for defects exceeding 4 × 6 cm.

**Figure 1:**
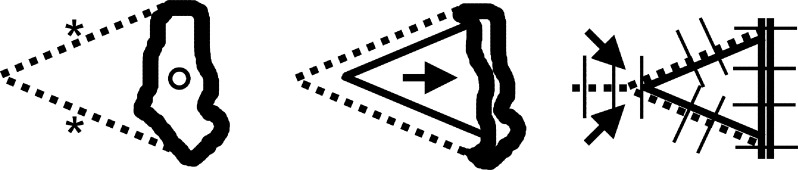
V-Y plasty. After a V-shaped incision (asterisks indicate incision lines, and the circle indicates defect), the margins are extended and sutured in a Y-shaped manner, thus elongating the tissue and allowing defect coverage.

Elliot, Bardsley, and colleagues^30^^,^^31^ have described closure of the RFFF donor site defect by using a transposed ulnar fasciocutaneous flap and a V to Y technique for the proximal forearm (evidence level IIc). Ahn et al^32^ and Bashir et al^33^ have used similar techniques; the former performed an elliptical design in the distal palmar forearm with the long axis oriented transversely parallel to the wrist. The donor defect is closed by a V-shaped flap, which is elevated as a fasciocutaneous flap based on the ulnar artery by V-Y advancement^32^^,^^33^ (evidence level IV). The authors have shown that this procedure eliminates the need for a skin graft, minimizes donor site morbidity, and significantly improves the aesthetic result (Fig 1).

**Figure 2:**
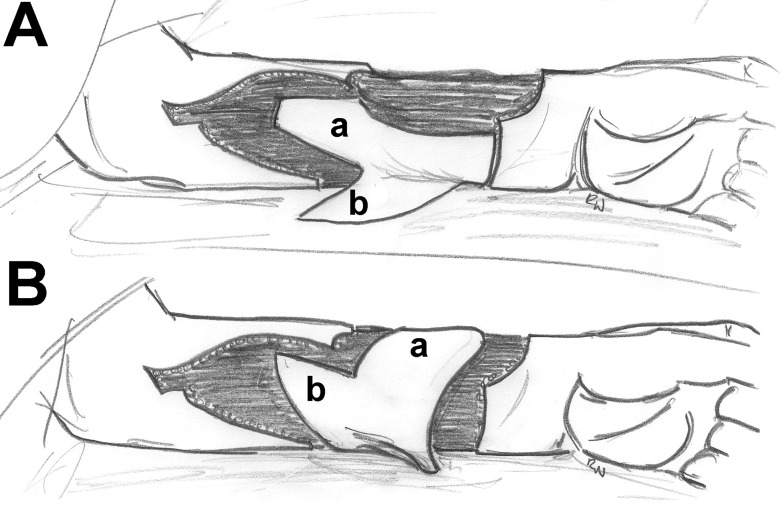
Bilobed flap technique. A: elevation of a large lobe (a) and a small lobe (b). B: rotation of the flaps; the radial forearm donor defect is covered with the large lobe, and the small lobe is used to repair the defect created by the large lobe. Drawing on the basis of Hsieh et al.^34^

Another technique aimed at avoiding a skin graft that would cause an additional donor defect has been described by Hsieh et al^34^ (evidence level IV), that is, RFFF donor site closure with a bilobed flap based on ulnar artery perforators. The defect sizes in this study range from 5 × 6 cm up to 8 × 8 cm, with an average defect of 47 cm^2^. The bilobed flap consists of a large lobe and a small lobe. After elevation, the flap is rotated, and the large lobe is used to cover the radial forearm donor defect, whereas the small lobe is used to repair the resultant defect from the large lobe (Fig 2).

In a case report (evidence level IV), Akyürek and Safak^35^ have described another local flap technique for the closure of the radial donor site: a so-called double-opposing rhomboid transposition flap, which is based on the existence of an oblique skin laxity in the distal forearm from the ulnar to the radial side. The authors postulate that their technique of direct closure with local flaps in one stage is superior to skin grafting with regard to donor site morbidity. Again, this technique of using local flaps is limited to small- to medium-sized defects (up to 6 × 4 cm) and, except for the study of Bardsley et al,^30^ not highly evidence based.

#### Tissue expansion

Tissue expansion is another alternative that can be used to diminish the donor site defect so that direct closure can be performed, but only if time permits. Tissue expanders are silicone envelopes with self-sealing injection ports. These devices are subcutaneously implanted near to the donor region, either pre- or posttransfer. Unfortunately, at least 2 operative stages are required for each of these options. The expander is implanted in a subcutaneous pocket that is located in the epifascial plane. Modern expanders are self-inflating absorbing body fluid from the surrounding tissue through osmotic action. The major advantage of this technique is the possibility of direct wound closure after flap harvesting without the need for a skin graft.^36^ However, a major disadvantage of this procedure is the frequent rate of complications (up to 40%),^37^ including an increased risk of infections, temporary tissue hypoxia caused by pressure peaks after saline installation and implant extrusion, and the delay of approximately 20 days prior to cancer surgery itself when used pretransfer.^38^ Studies focusing on decreasing donor site morbidity of the RFFF with tissue expanders are rare and do not exceed evidence level IV, despite their promising results in the case series cited previously.

#### Skin grafts

With regard to using an autologous or artificial skin graft instead of performing primary closure or a local flap plasty to close the RFFF donor site defect, various ideas have been proposed as to the best method to cover the full-thickness donor site defect. Most investigations into this topic are retrospective studies that examined aspects such as the rate of complete skin graft take, wound healing distributions, and aesthetical and functional impairments, especially with regard to hand function and health-related quality of life.

With regard to a high level of evidence, only 1 prospective RCT, namely, that by Sidebottom et al^39^ (actually evidence level Ib, but limited follow-up of approximately 50% leaded to a downgrading to level IIb), is available, which focused on the repair of the RFFF donor site with either full- or partial-thickness skin grafts. Sidebottom et al^39^ have found that, provided that an adequate graft is taken, full- and partial-thickness skin grafts have the same short-term and long-term outcomes in the repair of the RFFF donor sites. The same results with no significant differences concerning the aesthetic and functional outcome have been reported in the study from Zuidam et al^40^ (evidence level IIc) and from Ho et al^41^ (evidence level IIb), the latter have compared reconstruction with full-thickness skin graft (FTSG), STSG alone, and STSG overlying an acellular dermal matrix (AlloDerm).

Other available literature (all evidence level IV) reports better aesthetic results after closure of RFFF donor sites with FTSG, although these differences are marginal compared with STSG grafts.^42^^-^45 These studies emphasize better healing results, less wound breakdown and thus less morbidity, and improved aesthetic results after reconstruction with FTSG. Worthy of mention, the morbidity at the secondary donor site (eg, the abdominal wall or the groin) is reduced because of the option of its primary closure, in contrast to STSG donor sites, which need up to several weeks to heal completely.

To avoid creating a secondary donor site defect (in the case of an STSG, mostly the anterior thigh), Kawashima et al^46^ and Wolff et al^26^ (both evidence level IVc) have described the possibility of gaining an STSG from the RFFF itself for closure of its donor site. The de-epithelialized RFFF is then grafted into the oral cavity, where it reepithelializes in about 2 weeks. Both sets of authors suggest this technique as a possibility to reduce RFFF donor site morbidity without the risk of major complications. One must be aware of an approximately 15% shrinkage of the flap with slight cosmetic and functional impairment and the need for a 2-stage procedure.

#### Allogenic grafts

Allogenic grafts, instead of autologous skin grafts for coverage of the donor site defect of RFFF, did not become popular for a long time, until Rowe et al^47^ (with cadaveric acellular dermal matrix) and Wax et al^48^ (with AlloDerm) (both evidence level IIc) and recently Galego et al (with allogenic cultured epidermis)^49^ (evidence level IV) presented promising results. In their unfortunately small and not controlled patient groups, they report a longer wound healing period on the one hand, but on the other hand, the method avoids another donor site with similar aesthetic and functional outcomes. This has been confirmed by Ho et al^41^ in a high evidence level IIb study, who have compared FTSG with STSG, alone and combined with AlloDerm. AlloDerm is a processed, acellular, structurally intact dermal matrix derived from human cadaveric skin and can be used with or instead of STSG or FTSG. Its main advantage is that no second graft is necessary and therefore no secondary defect is produced. Wax et al^48^ have compared AlloDerm with conventional STSG and demonstrated that patients with allogenic dermis take between 12 and 16 weeks to recover completely whereas patients with an STSG are completely healed after 4 to 6 weeks^48^ (evidence level IIc). The prolonged healing period is a disadvantage, especially with respect to the special circumstances of already-weakened patients, as also shown in other studies.^50^ On the other hand, no extra skin graft for the coverage of the primary donor sites is needed, and the aesthetic result is judged to be marginally better in the AlloDerm group.

When artificial dermal templates are used in conjunction with STSG, for example Terudermis or Integra, the donor site is primarily covered with this artificial skin directly after flap raising. The collagen fibers of the artificial dermis resemble those of dermal tissue; host fibroblasts and capillaries become incorporated into the collagen sponge soon after its application to the wound. Dermal tissue is thereby regenerated as a result of the conversion of the collagen sponge into a pseudodermis, a neodermal tissue is formed within about 2 weeks, and this new tissue is then covered by an STSG. Accordingly, the artificial dermis has been designed with the intention of achieving the positive effects of a full-thickness skin graft, both mechanically and aesthetically, by combining the organized collagen matrix with an ultrathin STSG^19^^,^^51^^,^^52^ (all evidence level IV). Comparison of composite reconstruction with Integra artificial dermis compared solely with allogenic-graft-covered donor sites shows the faster healing within 4 to 6 weeks on the one hand, but the need for a 2-step procedure on the other hand.

Considering different flap raising options when using Integra, the suprafascial method seems to be superior to the subfascial method. Andreas et al^53^ pose the hypothesis that the well-vascularized deep forearm fascia ensures faster take of Integra, compared to the paratenon. Moreover, a greater amount of granulation tissue is formed under the silicone sheath that is liable for a superficial wound contraction^53^ (evidence level IV).

#### Vacuum-assisted closure

With regard to the topic of vacuum-assisted closure (VAC), marketed by Kinetic Concepts, Inc, San Antonio, Tex, Vidrine et al^54^ have demonstrated, in a level IIb-study, that STSG survival can be significantly improved by the use of subatmospheric pressure dressings. The VAC system has been suggested for use postoperatively as a bolster dressing over the STSG. Andrews et al have closely examined the procedure and show an increased incidence of small tendon exposures if the VAC bolster is not left in place for a minimum of 6 days^55^ (evidence level IIc). Vacuum-assisted closure therapy has also been used to deal with tendon exposure after failed skin grafting. Subatmospheric pressure dressings stimulate the growth of granulation tissue over the tendons and remove exudates from the wound, thereby contributing to improved graft adherence, which decreases donor site morbidity^55^ (evidence level IIc),^56^^,^^57^ (both evidence level IV).

**Figure 3:**
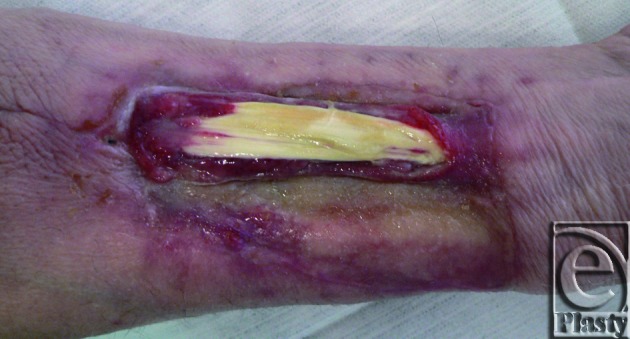
Split-thickness skin graft failure with exposure of the flexor tendons of the wrist.

In summary, with regard to the results of the closure of the donor site, a nonnegligible functional forearm and wrist range-of-motion morbidity is widely accepted to occur in the early postoperative period^58^ (evidence level IIc). The percentage of patients who experience more or less significant donor site complications ranges from 6% to 53% in the literature. Up to 16% of patients suffer from a restricted function of the donor forearm, and up to 28% complain of poor aesthetic results^30^^,^^59^ (both evidence level IIc). The most common complication is a failure of the skin graft, with exposure of the flexor tendons of the wrist (20%-33%; Fig 3), followed by nerve sensory disturbances (30%) and functional complications such as a reduction of wrist mobility as a result of the damage of parts of the Ramus superficialis of the radial nerve^15^^,^^30^^,^^34^^,^^56^^,^^59^^-^61 (evidence level IIc-IV).

However, only a moderate and acceptable incidence of long-term functional morbidity is associated with the radial artery removal^62^ (evidence level IVb) and the aesthetic morbidity after raising a fasciocutaneous RFFF is mostly negligible, whereas a higher incidence is observed with composite flaps. This is shown in well-conducted level IIc evidence-based studies, which focus directly on donor site outcome.^63^^-^67 Sardesai et al,^67^ for example, have evaluated long-term donor site morbidity of the RFFF qualitatively and quantitatively. They have shown that the operated arm exhibits a decreased dexterity of the hand, without change in wrist and forearm range of motion, but an increase in the range of motion of the little finger. In addition, a decrease in function and an increase in pain have been found by using the Michigan Hand Outcomes Questionnaire.

**Figure 4:**
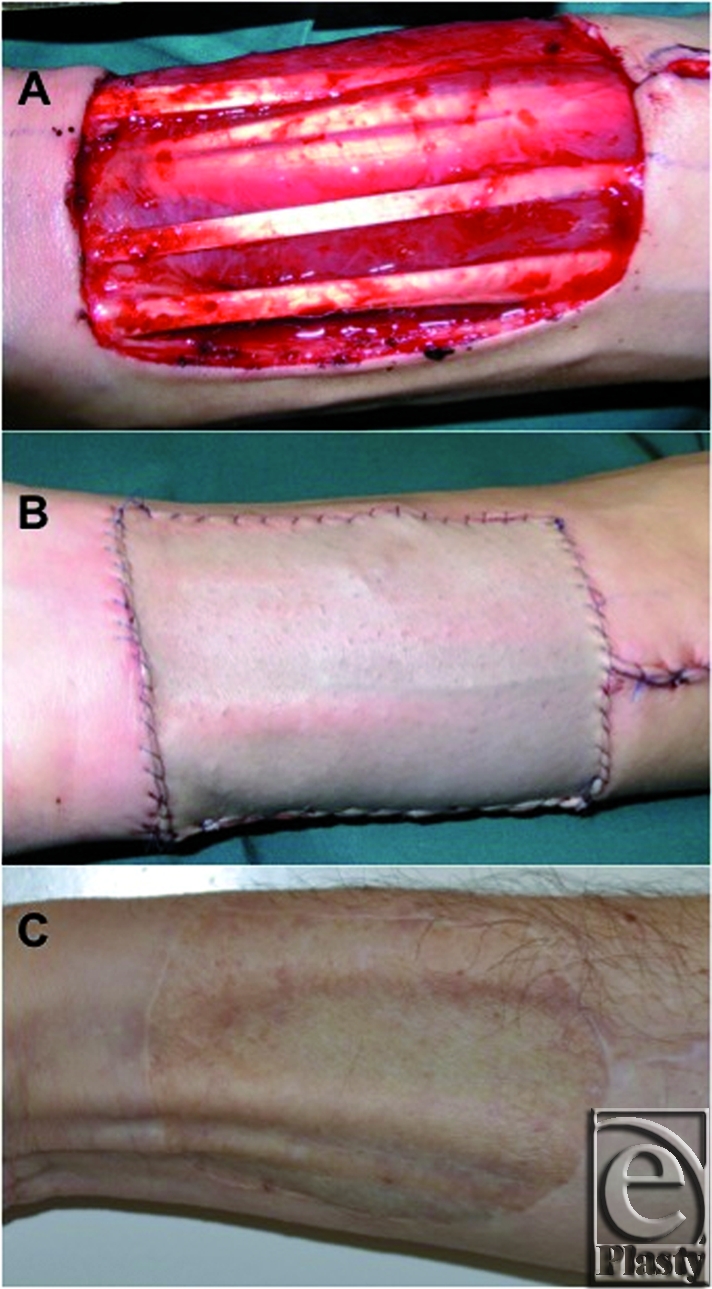
Coverage of a radial forearm free flap donor site with a split-thickness skin graft (STSG). A: Initial defect. B: After closure with an STSG. C: Long-term result after 3 months.

Nevertheless, Emerick and Deschler have demonstrated that only 1.9% of patients have severe complications requiring surgical intervention^68^ (evidence level IV); this is in accordance with the experience of the authors (Fig 4).

### Comparison of donor site morbidity to other free flaps

Progress in microsurgical procedures has led to the development of other free flaps for head and neck reconstruction that are comparable in their usage to the RFFF but with fewer donor site drawbacks. Predominately, the (thinned) anterolateral thigh cutaneous flap has become famous because of its comparable texture and versatility. It gives optimal results, either at the donor site or at the accepting site, and is easy to harvest. Furthermore, it can provide a long and constant pedicle of large caliber and can even be raised without the sacrifice of a main vessel. Studies that have compared both these flap types for head and neck reconstruction accentuate the lower donor site morbidity of the anterolateral thigh (ALT) flap compared with the RFFF.

Morrissey et al^69^ have not focused solely on the donor site but have carried out a prospective randomized trial in which the radial forearm flap (RFF) and its donor site morbidity have been prospectively compared with ALT free flaps for laryngopharyngectomy defects. They have found an increased free flap complication rate at the recipient site, with only a slight, but not significant, decreased flap donor-site morbidity. As such, they recommended the RFFF as the preferred flap for the reconstruction of laryngopharyngectomy defects^69^ (classified as level IIb, not Ib despite its clinical randomized controlled approach, but low-quality trial with only ALT:10/RFFF:9 patients in each group and only 60% long-term follow-up). As such, they recommend the RFFF as the preferred flap for reconstruction of laryngopharyngectomy defects.

An evaluation of donor-site morbidity in an evidence level IIb study by Huang et al^70^ (not classified as level Ib despite its clinical randomized controlled approach, as a retrospective analysis) of the RFFF and the thinned ALT cutaneous flap for reconstruction of tongue defects has shown that the latter is a viable substitute for the RFFF when reconstructing defects of the tongue. The results achieved are similar to those of the RFFF, and the donor-site morbidity is significantly decreased. The disadvantages of the RFFF include the unattractive scar in the forearm region, occasional numbness in the first 2 fingers, and the sacrifice of a major artery of the limb. In some patients, the donor-site scar of the forearm can act as a social stigma. In contrast, the ALT flap, after thinning, achieves the same results in reconstructing defects of the tongue without the associated donor-site morbidity. Its versatility in design and its long pedicle with a suitable vessel diameter are the characteristics of the ALT flap, and most importantly, the donor site in the thigh can be closed primarily in almost all patients with no functional deficit. This is in concordance to the authors' experience with the RFF and ALT flap for intraoral reconstruction^71^ (evidence level IIc), as we have recorded a significantly higher frequency of follow-up visits in the RFFF group as in the ALT patients (perioperative data analysis from 161 cases) and no wound healing disorder at the donor site of ALT patients compared with RFFF patients.

Another evidence level IIb study by de Vicente et al^72^ has highlighted the functional parameters of reconstructed hemiglossectomy defects in a controlled clinical trial. They have revealed no differences in mean speech intelligibility, tongue mobility, or deglutition mean scores between RFF and ALT flap.^72^

Two other evidence level IIc studies have also reported an improvement in donor site morbidity in the ALT group compared with the RFFF group.^73^ In about 30% of cases of the RFFF group, a persistent impairment in forearm movement and sensitivity alterations in skin graft area was noticed in 75% of patients. In the ALT group, the authors found only a transitory gait impairment in 1 of 25 patients. Furthermore, no clinical signs of circulatory disturbance were observed, and no sensory disturbance of the thigh was reported.^74^

Recently, our department evaluated the peroneal perforator flap for intraoral reconstruction of an ablative tumor defect of the floor of the mouth and tongue and cheek defects and found comparable results with either ALT or RFFF, with the limitation that no long-term follow-up was available at this early stage. We think that this flap is a good alternative in small- and medium-sized intraoral defects, particularly if direct closure at an inconspicuous donor site is desired^75^ (evidence level IV).

No further studies were found, which compared the RFFF donor site with other free flap donor sites directly.

## CONCLUSIONS

An obvious discrepancy between quality and quantity exists in the literature with regard to RFFF donor site morbidity. Practicing evidence-based medicine (EBM) means integrating individual clinical experience with the best available external clinical evidence. The difficulty is that EBM demands stronger evidence (eg, systematic review, RCTs) than traditional medicine has used, and these studies are rare in a field like plastic surgery.

The finding that 81.3% (n = 139) of all relevant contributions (n = 171, levels I-V) have an evidence level of III, IV, or V (Table 2) should serve as a warning to clinicians not to give the results of these papers exaggerated credit, especially in the light of the ever-growing influence of EBM.

If the best available evidence (levels I and II) is represented by only one-sixth of the available literature, as was the case in this analysis, the results are apt to be misinterpreted, and there is a latent danger of following non–evidence-based treatment strategies. This review is, however, limited because we have searched only the electronic databases, possibly leading to incomplete results. The results suggest that some treatment strategies are promoted with great self-confidence by certain authors with regard to the purported additional benefits for the donor-site morbidity of the RFFF, but that this is not based on solid scientific grounds.

Short-term donor site morbidity cannot be avoided in reconstructive surgery when using free flaps. Whenever switching to a free flap with less donor site morbidity than the RFFF is not possible, we conclude, on the basis of the reviewed literature, that small RFFF donor defects should be closed directly or with straightforward local advancement flaps if possible. The authors themselves mostly trust in a safe and reliable way of covering the RFFF donor site with autologous STSG after a classic subfascial flap raising technique. We consider it worth repeating that the paratenon of the exposed muscles should be left untouched to achieve an accurate wound bed and thus to avoid mobilizing and oversewing the surrounding muscle bellies.

Experimentation in a prospective and controlled way seems to be promising, as some articles have shown promising high-evidence–based results with closure by allogenic grafts, which offer equal or even better results than the use of autografts and, moreover, prevent the need for a secondary donor site. Further available methods, such as VAC therapy or tissue expanders, can be considered in extremely special situations.

This article should encourage researchers to carry out high-evidence clinical and experimental trials. When investigating new operative techniques for the closure of the RFFF donor site defect, a multicenter study with high volumes of RFFFs would be desirable to provide systematic research in decreasing donor site morbidity.

## Figures and Tables

**Table 1:** Hierarchy of strength of evidence for therapeutic decisions (modified according to Antes et al 8)^*a*^

Level	Studies of therapy, prevention, etiology, or harm
Ia	Systematic review (with homogeneity) of randomized controlled trial
Ib	Individual, randomized, controlled experimental or clinical trial
IIa	Systematic review of cohort studies
IIb	Individual cohort study (including low-quality randomized controlled trials; eg, <80% follow-up) and controlled experimental and clinical studies with no randomization
IIc	“Outcomes” research, eg, quality-of-life research, retrospective follow-up
IIIa	Systematic review (with homogeneity) of case-control studies
IIIb	Individual case-control studies
IV	Nonexperimental studies, such as cross-sectional trials, case series (and poor-quality cohort, follow-up, and case-control studies), case reports
V	Expert opinion without explicit critical appraisal or based on physiology, bench research, or “first principles”
(VI)	Not classified, because main focus on a topic other than donor site morbidity of the radial forearm flap

^*a*^A more detailed nomenclature can be found at http://www.cebm.net/index.aspx?o=1025.

**Table 2:** Studies focusing on donor site morbidity after transplantation of a free radial forearm flap^*a*^

Level of evidence	Literature
I) Ia)	None
Ib)	9^,^^10^^,^^14^
II) IIa)	None
IIb)	11^,^^39^^,^^41^^,^^54^^,^^69^^,^^70^^,^^72^^,^^76^
IIc)	15^,^^24^^,^^30^^,^^40^^,^^47^^,^^48^^,^^55^^,^^58^^,^^59^^,^^63^^-^67^,^^71^^,^^73^^,^^74^^,^^77^^-^80
III) IIIa)	None
IIIb)	44^,^^81^^,^^82^
IV)	12^,^^13^^,^^16^^-^23^,^^25^^,^^26^^,^^28^^,^^29^^,^^31^^-^38^,^^42^^,^^43^^,^^45^^,^^46^^,^^49^^-^53^,^^56^^,^^57^^,^^60^^-^62^,^^68^^,^^75^^,^^83^^-^166
IVa (number of participants >100)	n = 8
IVb (number of participants 20-100)	n = 43
IVc (number of participants <20)	n = 71
V)	27^,^^167^^-^180
(VI)	181^-^215

^*a*^ PubMed research 2011-10-05; key words: “donor site morbidity” and “radial forearm flap,” results: n = 188 plus articles (n = 18) found during research that were not identified by the database search: n = 206). Classification of the articles according to their levels of evidence. Classified as Evidence Levels I to VI as shown in Table 1.
